# Problematic barcoding in flatworms: A case-study on monogeneans and rhabdocoels (Platyhelminthes)

**DOI:** 10.3897/zookeys.365.5776

**Published:** 2013-12-30

**Authors:** Maarten P. M. Vanhove, Bart Tessens, Charlotte Schoelinck, Ulf Jondelius, D. Tim J. Littlewood, Tom Artois, Tine Huyse

**Affiliations:** 1 Laboratory of Biodiversity and Evolutionary Genomics, Department of Biology, University of Leuven, Leuven, Belgium; 2 Present address: Department of Botany and Zoology, Faculty of Science, Masaryk University, Brno, Czech Republic; 3 Research Group Zoology: Biodiversity & Toxicology, Centre for Environmental Sciences, Hasselt University, Diepenbeek, Belgium; 4 Aquatic animal health, Fisheries and Oceans Canada, Moncton, NB, Canada; 5 Department of Invertebrate Zoology, Swedish Museum of Natural History, Stockholm, Sweden; 6 Division of Parasites & Vectors, Department of Life Sciences, Natural History Museum, London, United Kingdom; 7 Department of Biology, Royal Museum for Central Africa, Tervuren, Belgium

**Keywords:** mitochondrial DNA, Monogenea, primer design, ribosomal DNA, Rhabdocoela, turbellarians

## Abstract

Some taxonomic groups are less amenable to mitochondrial DNA barcoding than others. Due to the paucity of molecular information of understudied groups and the huge molecular diversity within flatworms, primer design has been hampered. Indeed, all attempts to develop universal flatworm-specific COI markers have failed so far. We demonstrate how high molecular variability and contamination problems limit the possibilities for barcoding using standard COI-based protocols in flatworms. As a consequence, molecular identification methods often rely on other widely applicable markers. In the case of Monogenea, a very diverse group of platyhelminth parasites, and Rhabdocoela, representing one-fourth of all free-living flatworm taxa, this has led to a relatively high availability of nuclear ITS and 18S/28S rDNA sequences on GenBank. In a comparison of the effectiveness in species assignment we conclude that mitochondrial and nuclear ribosomal markers perform equally well. In case intraspecific information is needed, rDNA sequences can guide the selection of the appropriate (i.e. taxon-specific) COI primers if available.

## Introduction

Many biodiversity studies tend to focus on conspicuous fauna, ignoring the vast species diversity and ecological importance of less sizeable animals such as parasitic or meiofaunal taxa, including flatworms (Windsor 1998, Marcogliese 2004, Fonseca et al. 2010). To deal with the huge task of assessing their biodiversity and systematics, a variety of molecular-based methods have been proposed. These include a (phylo)genetic approach (Brooks and Hoberg 2001), DNA barcoding (Besansky et al. 2003) and amplicon-based next generation sequencing of environmental samples (Fonseca et al. 2010). DNA barcoding aims to use the sequence diversity of one or more uniform target genes to identify species (e.g. Stoeckle 2003, Hebert et al. 2004, Meusnier et al. 2008). Such a standardized approach is particularly promising in understudied taxa and for organisms where morphological identification is complicated, in case of heteromorphic generations, sexual dimorphism, a lack of suitable characters or, for example in parasites, the existence of larval stages that have not been characterized yet (Leung et al. 2009, Radulovici et al. 2010 and references therein). There are indications that the commonly used barcoding gene, cytochrome *c* oxidase subunit I (COI), is also suitable for tree reconstruction and molecular dating (e.g. for insects: Gaunt and Miles 2002, for digeneans: Brant and Loker 2009). Phylogenetic inference (or, for that matter, phylogeography or population assignment) is certainly a potential added value of COI barcoding. It does not, however, lie at its core (Moritz and Cicero 2004). While we do not intend to provide a review here on the pros and cons of barcoding, we completely agree with Besansky et al. (2003) that barcoding is not an end in itself. Rather, it is a sequence-based tool that may facilitate and accelerate identification of previously characterized species, e.g. from environmental samples, or that may assist in the detection of cryptic species (Vilas et al. 2005, Locke et al. 2010b, Nadler and Pérez-Ponce de Léon 2011, Jörger and Schrödl 2013).

It is important to consider the characteristics of the COI gene, warranting its common use as a barcoding gene. Being a mitochondrial gene, it has a maternal inheritance, lacks introns, undergoes no recombination, and primers are available for potentially much of the animal kingdom (Folmer et al. 1994, Hebert et al. 2003a). This supposed availability of universal primers is a core advantage, although truly universal applicability is questionable (Stoeckle 2003, Radulovici et al. 2010, Taylor and Harris 2012). Another asset is that intraspecific genetic distances are usually much lower than interspecific distances (Hebert et al. 2003b). Hebert et al. (2003a, b) demonstrated the wide applicability of COI, both at various taxonomic levels and across a range of taxa. What is the state-of-affairs, then, in barcoding abundant but inconspicuous animals such as those belonging to the parasitic realm or the meiobenthos? With the example of two species-rich and understudied groups of flatworms, rhabdocoels and monogeneans, we aim to evaluate the potential of COI for barcoding, and assess the potential of alternative ribosomal DNA markers.

### COI barcoding in monogenean and rhabdocoel flatworms: not a one-stop shop

**Figure 1.**
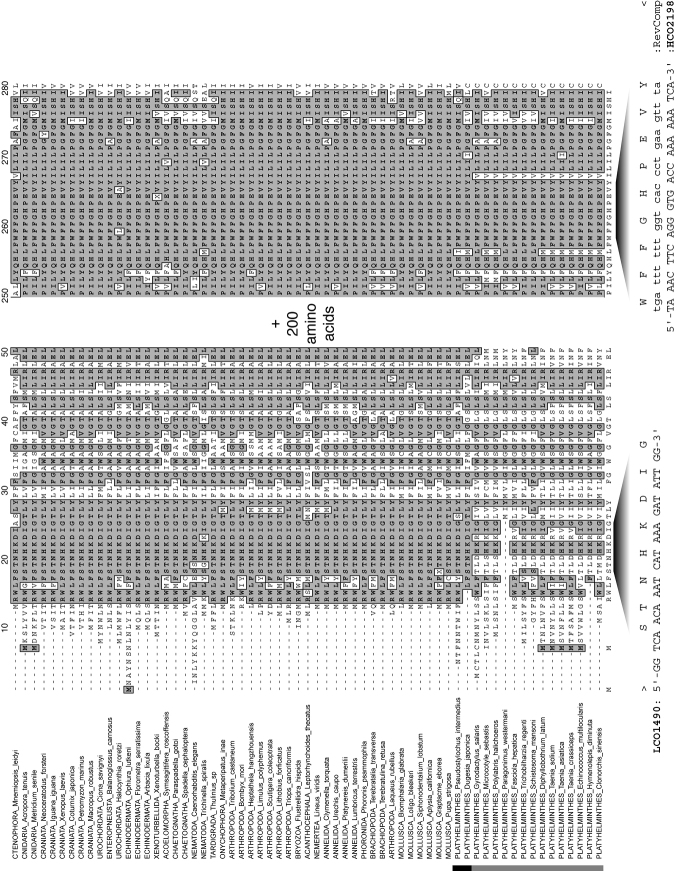
Fragments of an alignment of complete mitochondrial cytochrome *c* oxidase subunit I genes, as amino acids, for a diversity of metazoan taxa indicating the positions of the Folmer et al. (1994) primers. Shading indicates sequence identity to a consensus sequence calculated where > 50% taxa share amino acid identity at any position. All data available from MitoZoa database (Lupi et al. 2010). Vertical bar indicates platyhelminth taxa; black indicates ‘turbellarians’, grey indicates neodermatans.

The acquisition of COI markers for flatworms has opened up many new research avenues. COI data have proven useful in parasitic, meiofaunal or other flatworms (e.g. Elsasser et al. (2009) for guinea worms, Lázaro et al. (2009) for triclads, Sanna et al. (2009) for proseriates, Locke et al. (2010a) for diplostomid digeneans). However, the amplified COI fragment may lie outside the barcoding region (Moszczynska et al. 2009). The use of COI in flatworms can also entail amplification or sequencing problems (e.g. Larsson et al. (2008) for catenulids) or can simply be insufficiently explored (Casu et al. (2009) for proseriates). Moreover, COI amplification in flatworms may require the development of taxon-specific primers (Moszczynska et al. 2009, Sanna et al. 2009). Indeed, truly “universal” barcoding primers for flatworms are either lacking to date or underperform for certain groups (Littlewood 2008, Moszczynska et al. 2009). Within flatworms, there is considerable amino acid sequence variability in the region where Folmer et al. (1994) designed the “universal” COI primers, and flatworms seem radically different from other metazoans in amino acid content over the COI gene (Figure 1). Hence, it is easy to understand why it is difficult to find a set of primers that perform well for a wide range of flatworms. Indeed, despite their diversity, neither monogeneans nor rhabdocoels were well covered in papers central to the development of the barcoding idea, although these included flatworms. For example, Folmer et al. (1994) mentioned that the COI primers proved successful in a polyclad and a digenean flatworm, while Hebert et al. (2003b) scrutinized COI sequences from several families of cestodes, digeneans and triclads, but only included one monogenean family (Polystomatidae) and no rhabdocoels. The unavailability of truly ubiquitous PCR primers and conditions is suboptimal and undermines the use of COI as a barcoding marker universal to flatworms.

Monogenea is a species-rich group within the parasitic flatworms, a lot of the diversity of which remains unexplored. Indeed, only an estimated 2 200 – 5 000 species have been described (Hoberg 1997, Whittington 1998 and references therein), with a remaining 20 000 presently undescribed species (Whittington 1998). Monogenea mostly includes ectoparasites of cold-blooded amphibious or aquatic vertebrates, while some are endoparasites or infect aquatic invertebrates (Pugachev et al. 2009). Though clearly not as widespread in use as nuclear rDNA (Littlewood 2008) (see below), COI markers may offer high resolution for monogenean barcoding (Hansen et al. 2007). COI sequences are available from an increasing range of monogeneans, including representatives of Ancyrocephalidae, Capsalidae, Chauhaneidae, Chimaericolidae, Diclidophoridae, Discocotylidae, Diplectanidae, Diplozoidae, Gastrocotylidae, Gotocotylidae, Mazocraeidae, Microcotylidae, Plectanocotylidae, Polystomatidae and Pyragraphoridae (Telford et al. 2000, Jovelin and Justine 2001, Plaisance et al. 2008, Mladineo et al. 2009, 2013, Perkins et al. 2010, Li et al. 2011, Poisot et al. 2011, Schoelinck et al. 2012, Stefani et al. 2012, Zhang et al. 2012). COI sequences published for monogeneans are regularly positioned in the region amplified by the widely used ASmit primers (Littlewood et al. 1997). The COI gene region in question does not match the commonly used “barcoding fragment” *sensu* Folmer et al. (1994). Indeed, the 3’ primer (LCO1490) of the Folmer et al. (1994) set overlaps with the 5’ primer of ASmit (Asmit1) so that these fragments only overlap at primer-binding sites; the Folmer et al. (1994) fragment is ~ 459 bp and the adjoining ASmit fragment ~ 445 bp. Each of these fragments is less than a third of the complete COI gene. However, short barcoding fragments in general need not be problematic (Meusnier et al. 2008). Moreover, their length can of course be extended, e.g. in combination with a schistosomatid primer from Lockyer et al. (2003b) (e.g. up to *ca.* 580 bp in Vanhove (2012)).

Rhabdocoela is one of the most species-rich clades of free-living “turbellarian” flatworms with over 1 500 described species (Van Steenkiste et al. 2013). The suitability of the COI gene for DNA barcoding of rhabdocoels has not yet been explored. Since there are only four COI sequences from three species published in GenBank on 25 November 2013, a first goal of this paper is to obtain COI barcode data from different rhabdocoels by means of cloning. This approach is obviously not suited for large-scale applications, but can be used to identify possible contaminating factors and to establish a dataset of COI sequences that allows the development of new taxon-specific primers.

### The ribosomal DNA region and its use in species recognition in flatworms

Various fragments of the nuclear ribosomal DNA, like the genes for 18S, 5.8S and 28S rRNA, and the internal transcribed spacers ITS-1 and ITS-2, evolve at different rates, making them suitable for assessing genetic divergence at various levels (Hillis and Dixon 1991). The ribosomal RNA genes are rather conserved, allowing the design of primers for a wide range of taxa. Additional methodological advantages include the multicopy structure of rDNA (allowing amplification of little DNA template, e.g. in minute animals or museum specimens) and its concerted evolution, leading to low intraspecific variation (Hillis and Dixon 1991, Nieto Feliner and Rosselló 2007). The phylogenetic or taxonomic application of nuclear ITS rDNA is especially established in plants and fungi (Nieto Felliner and Rosselló 2007) but also popular in a wide range of animal taxa (e.g. Odorico and Miller 1997), including flatworms (e.g. Nolan and Cribb 2005, Brant and Loker 2009).

In monogeneans, various portions of the rDNA, and most often the spacer regions ITS-1 and ITS-2, are considered to adequately mirror differences between morphologically recognized species (Cunningham 1997, Matějusová et al. 2001, Meinilä et al. 2002, Ziętara and Lumme 2002). They are also useful in identifying cryptic species (e.g. Pouyaud et al. 2006). As a consequence, these sequence fragments are often included in species descriptions (e.g. Huyse and Malmberg 2004, García-Vásquez et al. 2007, 2011, Paetow et al. 2009, Paladini et al. 2009, 2010, 2011a, b, Přikrylová et al. 2009a, b, 2012a, b, Rokicka et al. 2009, Vaughan et al. 2010, Schelkle et al. 2011, Vanhove et al. 2011b, Ziętara et al. 2012, Řehulková et al. 2013). This goes especially for representatives of *Gyrodactylus* von Nordmann, 1832. As is often the case, ITS sequences in monogeneans display little (or no) intraspecific variation (Meinilä et al. 2002, Huyse et al. 2006, Přikrylová et al. 2012a, but see Vanhove et al. 2011b), precluding comparisons of interspecific *versus* intraspecific genetic diversity which is an important part of COI barcoding (Stoeckle 2003, Hebert et al. 2004). It is, however, unfortunate that many studies do not address potential intraspecific ITS diversity at all. By often sufficing with one or a few sequenced individuals per species, in general, ITS rDNA has been used for phylogenetic positioning in species descriptions rather than as a barcoding fragment.

In rhabdocoels, the 18S and 28S rDNA has been used extensively for phylogenetic analysis (Willems et al. 2006, Van Steenkiste et al. 2013). These gene fragments can be obtained very easily in rhabdocoels using universal primers. Most rhabdocoel morphospecies have unique 18S and 28S rDNA sequences, except for a few species of the genera *Microdalyellia* Gieysztor, 1938 and *Castrada* Schmidt, 1862. No data from the spacer regions are currently available.

From these examples, it is clear that the various portions of the nuclear rDNA region render it a versatile region for genetic approaches to systematics of both monogeneans and rhabdocoels. An additional advantage is the availability of primers that seem to be flatworm-universal (Lockyer et al. 2003a, Telford et al. 2003) or that are even applicable to a much wider range of organisms ranging from fungi to schistosome flatworms (White et al. 1990, Barber et al. 2000, Sonnenberg et al. 2008, Moszczynska et al. 2009). However, the use of rDNA markers for barcoding has rarely been formally tested in monogeneans and rhabdocoels. As a second goal of this paper, we will therefore formally test the usefulness of some candidate rDNA barcoding markers in selected cases in monogeneans and rhabdocoels. Whenever possible, we directly compare the performance of these rDNA markers to that of the traditional COI mitochondrial marker.

## Materials and methods

### Amplification success of COI in rhabdocoel flatworms

A total of 27 species of rhabdocoels(from 21 genera covering 15 out of the 35 rhabdocoel families) were collected from freshwater, marine or brackish water sites. Specimens were collected as described in Schockaert (1996) and stored in ethanol for subsequent molecular work. All specimens were studied alive and documented through drawings, pictures and videos. Specimen collection and sequence data are provided in Appendix 1.

DNA was extracted from whole or partial specimens using the QIAamp DNA micro kit (QIAGEN) according to the manufacturer’s instructions. Extracts were stored in duplicates (40 and 20 μl) for each specimen. The Folmer et al. (1994) region of the COI gene was amplified with the primers LCO1490 (5’-GGTCAACAAATCATAAAGTTGG-3’) and an adapted version of the HCO2198 primer (5’-TCATAGTAGCCSYTGTAAAATAAGCTCG-3’) using a touchdown PCR protocol [95 °C for 4 min, 2 ×(94 °C for 30 s, 58 °C for 30 s, 72 °C for 30 s), 2 × (94 °C for 30 s, 56 °C for 30 s, 72 °C for 30 s), 5 × (92 °C for 40 s, 45 °C for 40 s, 72 °C for 1min 15 s), 35 × (94 °C for 30 s, 51 °C for 40 s, 72 °C for 1 min 15 s), 72 °C for 10 min]. Illustra puReTaq Ready-To-Go PCR beads (GE Healthcare) were used to prepare reactions containing 3 μl DNA-extract, 0.2 μM of each primer and water for a final volume of 25 μl. PCR products were checked on 1.4% agarose gels stained with Gelred (Biotum Inc.), then were cleaned in Nucleofast 96 PCR plates (Macherey-Nagel, Düren). PCR products were then cloned using the TOPO TA for Sequencing Cloning Kit (Invitrogen) according to the manufacturer’s instructions. From each PCR product, eight colonies were picked and bidirectionally sequenced on an ABI3130XL Automated DNA sequencer (Applied Biosystems, Hitachi). Sequences were visually inspected and assembled in Geneious Pro v5.7.5 (Biomatters Ltd).

To check for possible contamination we first submitted all sequences of each clone to BLAST search on the NCBI website (http://www.ncbi.nlm.nih.gov). To further identify sequences that did not have a strong match in GenBank we aligned them to a reference dataset of the Folmer et al. (1994) region COI sequences of possible contaminants such as known food items. This reference dataset was constructed with sequences collected from GenBank (see Appendix 2) and included the following taxa: Platyhelminthes, Arthropoda, Gastropoda, Bivalvia, Nematoda, Cnidaria. Sequences were aligned in ClustalX v2 (Larkin et al. 2007). A Neighbour-Joining (NJ) tree based on Kimura 2-parameter (K2P) (Kimura 1980) distances was calculated in MEGA5 (Tamura et al. 2011).

### Test cases for barcoding with ribosomal and mitochondrial markers

Three test cases were analyzed to demonstrate the potential of different markers for DNA barcoding in Monogenea and Rhabdocoela. The first consisted of 33 species from four genera from the monogenean family Diplectanidae infecting groupers from the Indo-Pacific (from Schoelinck 2012). This dataset contained 117 sequences of the COI gene and the nuclear 28S rDNA region (see Appendix 3). A second test case consisted of eight species from the monogenean genus *Gyrodactylus* (from Vanhove 2012). The species included are parasites of Balkan freshwater gobies (Vanhove et al. 2012, 2013). This dataset contained 35 sequences of the ITS-1 – 5.8S rDNA – ITS-2 rDNA region, 17 sequences of the COI gene and 38 sequences of the cytochrome *c* oxidase subunit II gene (COII) gene (see Appendix 3). The latter, of which over 600 bp was amplified by the primers developed by Bueno Silva (2011) is a promising additional marker for *Gyrodactylus* (Vanhove 2012). These degenerate primers can be optimized for other monogenean families (W.A. Boeger and M. Bueno Silva, personal communication), though there does not seem to be a single COII protocol which is generally suitable for non-gyrodactylid monogeneans. As a third case, we reanalyzed the nuclear 18S and 28S data from the analysis of Van Steenkiste et al. (2013) for the rhabdocoel genus *Gieysztoria* Ruebush and Hayes, 1939 (see Appendix 4). Additionally, we sequenced the ITS-1 – 5.8S rDNA – ITS-2 rDNA region from the same specimens (sequences deposited in GenBank under accession numbers KF953866–KF953883; see Appendix 4).

The K2P-distance model (Kimura 1980) was used to calculate sequence divergences between and within species. Histograms of intra- and interspecific distance frequencies were reconstructed in R v2.15.2 (R Core Team 2012) using scripts made available by G. Sonet (RBINS – Brussels, Belgium). For the monogenean test cases (test case 1 and 2), the proportion of correctly identified specimens was estimated with the program Species Identifier using the best match (BM) and best close-match (BCM) criteria of Meier et al. (2006). The threshold used in the BCM analysis was the “best compromise threshold” (BCTh) based on cumulative distribution curves of intra- and interspecific K2P-distances calculated in R (Lefébure et al. 2006). Species represented by a single sequence in the dataset were removed as they will generate incorrect identifications under the BM and BCM criterion because there are no other conspecifics in the dataset. For this reason, the BM and BCM criterion was not used on the rhabdocoel dataset (test case 3) where there are many species represented by a single sequence.

## Results

### Amplification success of COI in rhabdocoel flatworms

**Figure 2.**
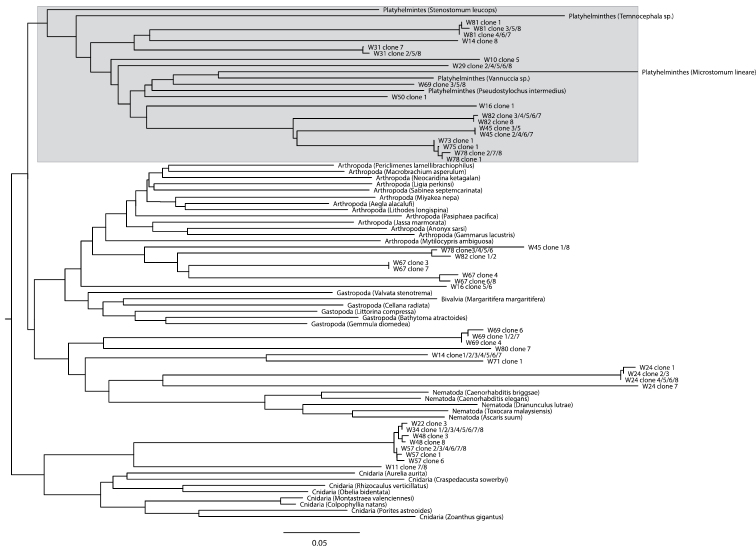
Neighbour-Joining tree based on Kimura 2-parameter (Kimura 1980) distances for COI DNA sequences for 100 clones from 27 rhabdocoel species, five flatworm COI sequences available from GenBank and 31 reference COI sequences from taxa that are potential food sources for rhabdocoels. The clade with platyhelminth sequences is indicated in gray.

A BLAST search of the 169 clones that could successfully be sequenced showed that contamination originated both from external DNA sources (*Homo sapiens*, *Bos taurus* – the latter possibly stemming from liver fed to flatworm cultures, or from bovine serum albumin used in the laboratory) and from food items eaten by the worms (Arthropoda, Annelida, Rotifera, Cnidaria, Ciliophora). Most rhabdocoels are so small that DNA has to be extracted from whole animals, which potentially results in the amplification of food items present in the animal. Only two sequences could be identified by BLAST as belonging to flatworms. This is, however, not very surprising given that there is currently only one rhabdocoel COI sequence overlapping with the Folmer et al. (1994) region available in GenBank. Only by aligning the sequences that did not have a significant BLAST hit (accounting for 100 of the 169 clones) to a reference dataset containing published COI sequences from different flatworm species and some possible food items (Appendix 2), were we able to identify a clade of flatworm sequences (Figure 2). From the original 169 clones that were sequenced, only 19 sequences, belonging to 13 (out of a total of 27 investigated) species were identified as belonging to Platyhelminthes. Genetic diversity among these newly identified COI sequences from 13 rhabdocoel species is high (average pairwise K2P-distance = 0.284).

### Test cases for barcoding with ribosomal and mitochondrial markers

**Figure 3.**
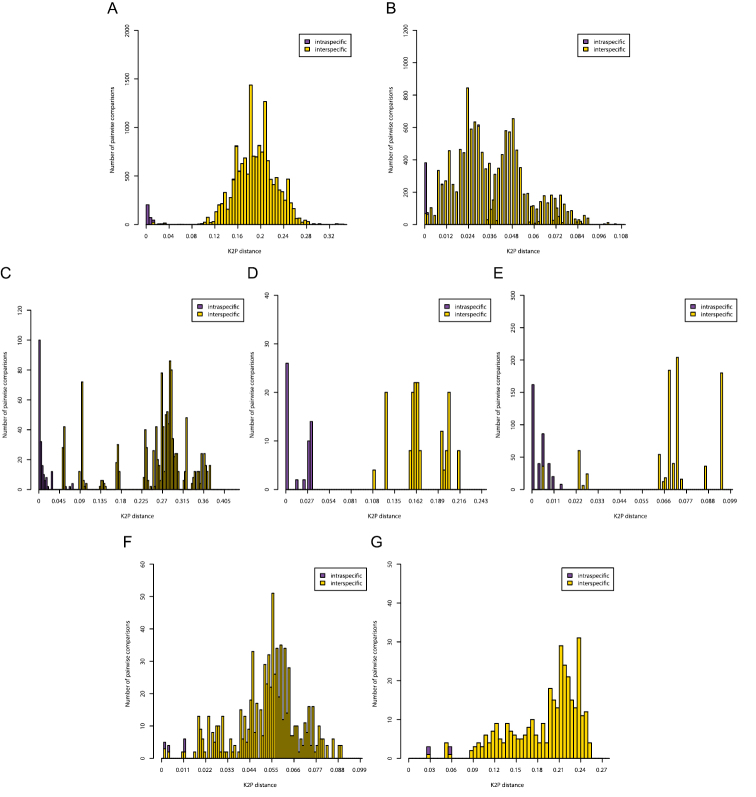
Pairwise distance (K2P) distributions of intra- and interspecific sequence divergences for the COI gene in Diplectanidae (**A**), 28S rDNA region in Diplectanidae (**B**), the COII gene in *Gyrodactylus* (**C**), the COI gene in *Gyrodactylus* (**D**), the ITS rDNA region in *Gyrodactylus* (**E**), the 28S rDNA region in *Gieysztoria* (**F**) and the ITS – 5.8S – ITS2 rDNA region in *Gieysztoria* (**G**).

**Figure 4.**
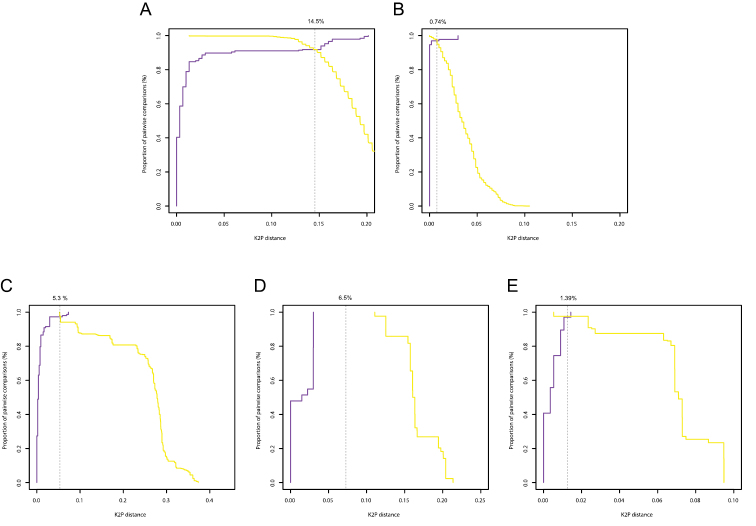
Optimum threshold defined by the intersection between the cumulative frequency distribution curves of the intraspecific (purple) and the interspecific (yellow) pairwise distances for the COI gene in Diplectanidae (**A**), 28S rDNA region in Diplectanidae (**B**), the COII gene in *Gyrodactylus* (**C**), the COI gene in *Gyrodactylus* (**D**), the ITS rDNA region in *Gyrodactylus* (**E**).

Histograms of intra- and interspecific K2P-distances are given in Figure 3. Only for the COI gene of *Gyrodactylus* there was a clear barcoding gap (3-11%). In all other cases there was overlap between the distribution of intra- and interspecific K2P-distances. In the Diplectanidae dataset (test case 1) the BCTh values were 14.5% for COI and 0.74% for 28S (Figure 4). In *Gyrodactylus* (test case 2) the BCTh was 5.3% for COII, 6.5% for COI and 1.39% for the entire ITS-1 – 5.8S – ITS-2 fragment (Figure 4). Alignment of ITS fragments needs to take into account many indels, even in this dataset with closely related species.

In Diplectanidae, the identification success for the 33 species was high for both COI and 28S (Table 1). In the COI dataset there was only a single incorrect identification. In the 28S dataset there were no misidentifications, but nine identifications were ambiguous because *Diplectanum nanus* Justine, 2007 and *Diplectanum parvum* Justine, 2008 share an 28S sequence despite an average COI divergence of 1.9%. In *Gyrodactylus*, the identification success of the eight species was 100% with all three markers.

**Table 1.** Identification success, with best compromise threshold (BCTh) values used, as determined via the best match (BM) and best close-match (BMC) criteria.

Dataset		Threshold (%)	Correct	Ambiguous	Incorrect	No match closer than threshold
Diplectanidae COI	BM	-	116 (99.15%)	0	1 (0.85%)	-
	BCM	14.50%	116 (99.15%)	0	1 (0.85%)	0
Diplectanidae 28S	BM	-	108(92.3%)	9 (7.69%)	0	-
	BCM	0.74%	107 (91.45%)	9 (7.69%)	0	1 (0.85%)
*Gyrodactylus* COII	BM	-	38 (100%)	0	0	-
	BCM	5.30%	38 (100%)	0	0	0
*Gyrodactylus* COI	BM	-	15 (100%)	0	0	-
	BCM	6.50%	15 (100%)	0	0	0
*Gyrodactylus* ITS	BM	-	35 (100%)	0	0	-
	BCM	1.39%	35 (100%)	0	0	0

## Discussion

In order for COI to function as a widely used barcoding marker, ideally primers should be available allowing amplification of the gene under standard conditions for a wide range of taxa. For rhabdocoels, a taxon where the acquisition of COI data is clearly lagging behind, our results show that using universal COI barcoding primers is problematic. Universal primers seem to amplify non-rhabdocoel DNA much more efficient. This leads to contamination problems where several sequences are present in the PCR product and the resulting chromatogram becomes difficult to interpret. Problems with limited success of universal barcoding primers and with contamination by associated fauna are known from other animals as well, e.g. marine free-living nematodes (Derycke et al. 2010). Because of the high variation within the obtained rhabdocoel COI sequences it was also not possible to use this dataset to develop internal rhabdocoel-specific primers. Efforts to establish COI barcoding protocols in rhabdocoels should therefore probably focus on smaller taxonomic entities within this taxon.

### What can alternative markers offer?

Though less acute than in rhabdocoels, amplification success in our view is the biggest limitation to a wider use of COI barcoding in monogeneans as well. Despite the recent increase in published monogenean mitogenomes (e.g. Huyse et al. 2007, 2008, Park et al. 2007, Plaisance et al. 2007, Perkins et al. 2010, Kang et al. 2012, Zhang et al. 2011, 2012), universal COI barcoding primers have not yet been developed for monogeneans, let alone for flatworms in general. While advances in mitogenomics will hopefully facilitate the development of primer combinations for additional molecular markers, the mitochondrial genomes seem variable to such an extent that such primers will often forcibly be taxon-specific (as exemplified for the Folmer et al. (1994) region of COI in Figure 1). Hence, we agree with McManus et al. (2004) that the “post-genomic era” has clearly not dawned yet for parasitic flatworms, or, we suggest, flatworms in general. Moreover, as barcoding should be a user-friendly technique, ideally suitable also to the non-molecularly trained, relying on a set of taxon- or marker-specific protocols does not seem an ideal way forward. The nuclear rDNA region, including the ITS, is a better candidate in terms of widely suitable and versatile molecular markers. Their continued (and increased) use would of course exacerbate the existing “bandwagon effect” (e.g. Nieto Feliner and Rosselló 2007), but this need not be a problem. Indeed, any barcoding approach is only as good as the resulting available datasets. This limitation is evident even in better-studied taxa like fishes, for which barcoding efforts are considerable (Ward et al. 2009, Taylor and Harris 2012). While widely used COI barcoding primers are available (Ward et al. 2005), Vanhove et al. (2011a) demonstrated that for gobies, even within Europe, mitochondrial 12S and 16S rDNA yielded a bigger reference dataset and were hence better suited for phylogenetic assignment of unidentified species. Needless to say, similar problems exist for helminths (e.g. Palesse et al. 2011: philometrid nematodes). In contrast to mitochondrial markers, rDNA sequences can very easily be retrieved from both Monogenea and Rhabdocoela. The number of monogenean or rhabdocoel flatworms covered by rDNA sequences presently far outnumbers those for which COI data are available. This is clearly illustrated by the number of sequences available in GenBank (on 29 November 2013): a) Rhabdocoela: 233 18S, 144 28S, 0 ITS-1, 0 ITS-2 and 4 COI and b) Monogenea: 2298 rDNA and 1250 COI sequences, of which one-third from only three species: *Gyrodactylus salaris* Malmberg, 1957, *Gyrodactylus arcuatus* Bychowsky, 1933 and *Gotocotyla sawara* Ishii, 1936. Despite the importance of reference datasets, this in itself may not be an argument to favour rDNA over COI as a barcoding marker. The information content of the respective markers should be compared.

Our analysis of the distributions of intra- and interspecific K2P-sequence divergence shows that, in most cases, there is no clear DNA barcode gap in either COI or rDNA. However, since coalescent depths are known to vary among species, such overlap is to be expected and has indeed been reported in many other taxa (see, for example, Wiemers and Fiedler 2007, Virgilio et al. 2010, Breman et al. 2013). As Collins and Cruickshank (2013) recently argued, this lack of a barcode gap does not necessarily mean that these markers are not suited for species level identifications because there might still exist a “local barcode gap”.

Our analyses of Diplectanidae and *Gyrodactylus* show that both rDNA and mitochondrial markers can be highly effective for species identification. It is clear that the slower evolutionary rate of the rDNA markers does not necessarily make them less suited for DNA barcoding. We therefore suggest, also for monogeneans, to continue using rDNA markers. Both the 28S and ITS region could potentially be used as barcode marker. Our analysis of *Gieysztoria* shows that the faster evolving ITS region does not necessarily show a more pronounced DNA barcode gap (Figure 3). The choice between both markers should therefore be based on the species that need to be identified. The 28S region can be aligned more easily between distantly related species than the ITS region. Indeed, alignment problems have been reported for ITS in several monogeneans (Desdevises et al. 2000, Poisot et al. 2011). This limits the applicability of this marker to phylogeny reconstruction and genetic distance calculation, but does not preclude its use in species recognition. Indeed, while different rates of concerted evolution cause difficulties in phylogeographic analyses (Harris and Crandall 2000), various homogenization mechanisms most often lead to clear distinctions at the species level (Odorico and Miller 1997). Likewise, while the non-coding nature of ITS allows substantial length differences possibly precluding reliable alignment, this is of less concern when working with closely related species (Nieto Felliner and Rosselló 2007).

Yet, the slower evolving rDNA genes might not be suited to discriminate between very recently diverged species. More conservative than ITS-1 and ITS-2, they are more suitable for deeper phylogeny reconstruction than for example the detection of cryptic species. This was evident in our analysis of Diplectanidae where *Diplectanum nanus* and *Diplectanum parvum* shared a 28S rDNA sequence while their difference amounted to a maximum of 3.2% in COI. However, in most cases, the 18S and 28S rRNA genes can also differentiate among closely related monogenean and rhabdocoel species (e.g. Gilmore et al. 2012, Van Steenkiste et al. 2013). There are exceptions to this rule (e.g. Přikrylová et al. 2013 for identical 18S sequences in recently diverged *Gyrodactylus* species), which is not surprising given the extensive divergence rate variation throughout Monogenea (Olson and Littlewood 2002).

Unfortunately, because rDNA has exclusively been used in a phylogenetic setting in Rhabdocoela, there is too little information about intraspecific distances to formally test its use as a barcoding marker for rhabdocoels. We suggest that further efforts to establish a DNA barcoding protocol focus on the 28S rDNA region instead of the ITS region because the overlap between intra- and interspecific distances is not smaller in the faster evolving ITS, and because the ITS region is very difficult to align, even between closely related sequences.

### The way forward

Given the different applicability of the various markers, we suggest the approach offered by Moszczynska et al. (2009) for digeneans would be a suitable way forward in our target organisms as well. Widely applicable rDNA primers could be used in an initial, prospective step. Once the organisms in question have been assigned to a lower taxonomic rank, appropriate COI primers for the taxon can be selected, when sequences from a faster-evolving and mitochondrial marker are desired, for example to assess for recently diverged or cryptic species. This is, of course, highly dependent on the availability of such COI primers, which we showed to be problematic in certain taxa. Although this differs from the “classical” approach of barcoding with a standard marker and protocol, a combined use of COI with portions of the nuclear rDNA region fulfills most promises of DNA barcoding in monogeneans and rhabdocoels.

## Appendix 1

Supplementary table 1. (doi: 10.3897/zookeys.365.5776.app1) File format: Microsoft Excel file (xls).

**Explanation note:** List of clones sequenced in this study with species on which PCR was performed.

## Appendix 2

Supplementary table 2. (doi: 10.3897/zookeys.365.5776.app2) File format: Microsoft Excel file (xls).

**Explanation note:** Reference sequences downloaded from GenBank with accession numbers.

## Appendix 3

Supplementary table 3. (doi: 10.3897/zookeys.365.5776.app3) File format: Microsoft Excel file (xls).

**Explanation note:** List of species and number of sequences from each marker used in the monogenean test cases.

## Appendix 4

Supplementary table 4. (doi: 10.3897/zookeys.365.5776.app4) File format:Microsoft Excel file (xls).

**Explanation note:** List of species and GenBank accession numbers from the genus *Gieysztoria*.
